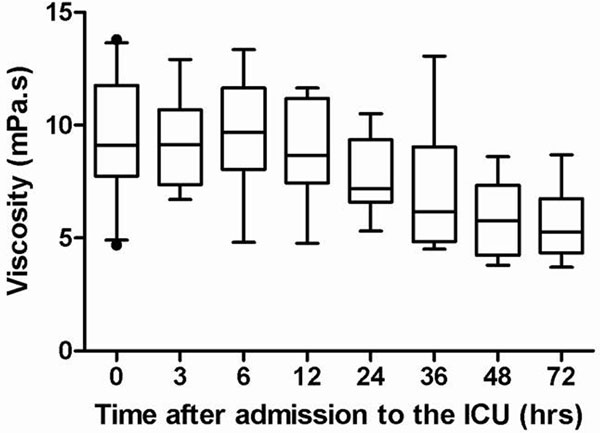# Effects of viscosity on cerebral blood flow after cardiac arrest

**DOI:** 10.1186/cc12250

**Published:** 2013-03-19

**Authors:** L Bisschops, G Pop, S Teerenstra, J Van der Hoeven, C Hoedemaekers

**Affiliations:** 1 Radboud University Nijmegen Medical Centre, Nijmegen, the Netherlands

## Introduction

After cardiac arrest, microcirculatory reperfusion disorders develop despite adequate cerebral perfusion pressure. Increased blood viscosity strongly hampers the microcirculation, resulting in plugging of the capillary bed, arteriovenous shunting and diminished tissue perfusion. The aim of the present study was to assess blood viscosity in relation to cerebral blood flow in patients after cardiac arrest.

## Methods

We performed an observational study in 10 comatose patients after cardiac arrest. Patients were treated with hypothermia for 24 hours. Blood viscosity was measured *ex vivo *using a Contraves LS300 viscometer. Mean flow velocity in the middle cerebral artery (MFVMCA) was measured by transcranial Doppler (TCD) at the same time points.

## Results

The median viscosity on admission was 9.12 (8.19 to 11.19) mPa. second, and remained stable at 3 and 6 hours after admission. From 6 hours after admission, viscosity decreased significantly to 3.66 (3.12 to 4.04) mPa.second (*P <*0.001). Median MFVMCA was low (27.0 (23.8 to 30.5) cm/second) on admission, and significantly increased to 63.0 (51.0 to 80.0) cm/second at 72 hours (*P <*0.001). There was a significant association between viscosity and the MFVMCA (*P *= 0.0019). See Figure 1.

## Conclusion

Viscosity decreases in the first 3 days after cardiac arrest and is strongly associated with an increase in cerebral blood flow. Since viscosity is a major determinant of cerebral blood flow, repeated measurements may guide therapy to help restore cerebral oxygenation after cardiac arrest.

**Figure 1:**